# A Computer Vision Method for Finding Mislabelled Specimens Within Natural History Collections

**DOI:** 10.1002/ece3.71648

**Published:** 2025-07-13

**Authors:** Jack D. Hollister, Geoff Martin, Xiaohao Cai, Tammy Horton, Owain Powell, Mark Sterling, Glory Turnbull, Ben W. Price, Phillip B. Fenberg

**Affiliations:** ^1^ Natural History Museum London UK; ^2^ School of Ocean and Earth Science, National Oceanography Centre University of Southampton Southampton UK; ^3^ National Oceanography Centre Southampton UK; ^4^ School of Electronics and Computer Science University of Southampton Southampton UK

## Abstract

Natural history collections are essential for biodiversity and evolution research and for studying biotic responses to global change. However, the numbers of specimens within natural history collections pose management challenges. Reduced funds, declining taxonomic training and expanding collections can lead to mislabelled or missing specimens. This highlights the need for innovative and non‐destructive methods of taxonomic verification for specimens in large collections. While genetic analyses offer precise verification, they are resource‐intensive and less effective on degraded DNA from older specimens, with risks of damage to smaller specimens. Computer vision can automate tasks such as species‐level verification and morphological examination, though these techniques have yet to be incorporated and utilised by natural history collections for such management tasks. Digitisation initiatives, such as those at the Natural History Museum (NHM), London, have gained momentum in recent years, converting specimens to digital formats and enhancing global accessibility. Here, we describe a computer vision pipeline applied to the digitised British and Irish Lepidoptera collection at the NHM. Specifically, our pipeline identifies specimens that do not match their labelled species status. The pipeline was executed for 100 runs for the Butterfly and Moth datasets, resulting in 99,350 out of 350,208 specimens (28.37%) being flagged at least once. We attribute a portion of these as pipeline errors, given the likelihood of some mislabelled specimens within training datasets. However, specimens flagged consistently across > 80% of pipeline runs are likely mislabelled within the collections. Taxonomic experts visually examined 210 such specimens, finding 145 to be incorrectly labelled in the collection or the NHM data portal. Additionally, 30 specimens were sent for genetic verification to confirm species‐level identification. This synergy of computer vision and genetic‐based species identification enhances the accuracy and efficiency of managing natural history collections, preserving their value for future generations.

## Graphical abstract

To enhance the management of vast natural history collections facing mislabelling challenges, we developed a computer vision pipeline to identify specimens whose images do not match their labelled species status. Applied to the digitised British and Irish Lepidoptera collection at the Natural History Museum, the computer vision pipeline flagged potential mislabelled specimens, which were then confirmed by taxonomic experts and genetic analysis. This integration of computer vision with traditional verification methods improves the accuracy and efficiency of collection management, preserving their value for future research.

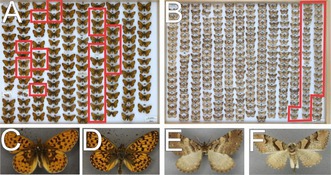

## 1 Introduction

Natural History Collections (NHCs) are essential datasets for much of modern‐day ecology and evolution research (Popov et al. 2021), including as baseline data for documenting biotic response to global change (Wilson et al. 2023). With the recent push towards massive digitisation efforts by natural history museums, NHCs have become ever more accessible to researchers, educators and the general public. However, with more researchers accessing large digitised NHCs (Hardy et al. 2023), it becomes increasingly important to ensure that specimen label information is accurate (e.g., species name). But finding and correcting specimen label errors within large NHCs is resource‐intensive and time‐consuming.

The curation, upkeep and maintenance of access to NHCs are major challenges for museums. For example, funding and staffing have not kept pace with the expansion of collections, leading to shortcomings in management and care of these critical resources (Paknia et al. 2015). Adding to these challenges is the decreasing reliance on traditional morphological identification methods, due to a decline in the number of taxonomic specialists, resulting from an ageing expert base and a lack of incoming specialists. As expertise in visual morphological identification decreases, maintaining the accuracy and integrity of these extensive collections becomes increasingly challenging (Godfray 2002; Bik 2017). One of the unavoidable consequences of these challenges is the general reduction in time and expertise dedicated to the maintenance of collections and specimen label information, including the time needed to properly curate an increasing number of new specimens deposited at museums. This can result in out‐of‐date taxonomic information, missing or illegible labels, incorrect species identification and/or errors in database entry.

The exact number of mislabelled specimens or other label errors is hard to define and will be collection dependent. Some groups have been well studied and kept up to date, with rich histories and knowledge associated with them (Salmon 2000), while others can be severely lacking in knowledge and expertise. For instance, a recent study found that 58% of tropical plant specimens they reviewed were misidentified and estimated that 50% of all tropical plant specimens are likely to be mislabelled within NHCs (Goodwin et al. 2015). The authors indicate that this is due to the large influx of specimens deposited since 1970 and the lack of taxonomic experts with the knowledge base required to classify them. Regions in the tropics and developing countries, characterised by high biodiversity and complex environments, have historically been under sampled, leading to a lower knowledge base associated with them compared to other areas (Moura and Jetz 2021). NHCs also hold the exciting possibility of containing undiscovered species (Parsons et al. 2022). These species may be hidden under incorrect labels or overlooked because of their scarcity and strong morphological resemblance to known species. The minor differences distinguishing these species can be difficult to detect through standard examination, especially when they are closely related (i.e., cryptic species).

All the above underscores the need for accurate identification methods in collections, whether for curatorial purposes or biodiversity discovery. Modern methods for species identification, like genetic analysis, offer accuracy but come with high resource demands (Shendure et al. 2017). Applying genetic analysis to entire larger collections could lead to astronomical expenses and extensive time requirements. Moreover, the DNA in historical or dried specimens is often degraded, thus providing less information than that of fresh or well‐preserved samples and requires more robust genetic‐based examinations (Marinček et al. 2022; Molbert et al. 2023; Rayo et al. 2024). Furthermore, many historical specimens are deemed to be too important for destructive sampling. As such, extracting DNA from these specimens is not always viable.

In addition, many museums have embarked on the mass digitisation of their collections, a step that serves multiple purposes (Hardy et al. 2023). Digitisation not only preserves the physical integrity of specimens but also allows them to become readily available for researchers across the globe, fostering wider collaboration and analysis, and significantly enriching our understanding of biodiversity and natural history.

In parallel to the mass digitisation of collections is the major advancement of artificial intelligence (AI) which has the potential to revolutionise the way collections are analysed and utilised (Groom et al. 2023). In particular, computer vision (CV) methods can be used for rapid species identification (Hollister et al. 2022), pattern recognition and morphological analyses (Hollister et al. 2023). The careful coupling of CV with digitised NHCs can bring unprecedented efficiency, accuracy and speed to species identification, which is a core component of collections management and museum‐based research.

Beyond verification, CV opens a myriad of possibilities for diverse research projects, ranging from tracking phenotypic changes with temperature (Wilson et al. 2023) to understanding complex ecological interactions (Johannes et al. 2024). The integration of CV into natural history research could not only streamline labour‐intensive processes of verifying the integrity of the organisation of collections but also pave the way for innovative methods of exploring and interpreting the vast datasets these collections represent. As AI continues to evolve, it promises to unlock new dimensions of knowledge and collaboration in the study of biodiversity (Karbstein et al. 2024; Borowiec et al. 2022; Seeland et al. 2019; Wäldchen and Mäder 2018). A CV‐based system or assistive tool could help alleviate some of the burden of managing large NHCs by scanning large collections of digitised specimens at high speeds, highlighting discrepancies leading to a streamlined and more accurate verification process.

One of the first massive digitisation projects was the ‘iCollections’, a programme undertaken by the Natural History Museum (NHM), London to digitise its collections of British and Irish butterflies (Paterson et al. 2016). The data captured includes species name, georeferenced location, collector and collection date, along with a digital image of each specimen and a scale for size reference. This initiative is part of a broader NHM programme to digitise its vast collections, comprising approximately 80 million specimens and objects. The iCollections data have been used to address various scientific questions, such as how climate warming might affect species distribution, phenology and body size (Wilson et al. 2023; Fenberg et al. 2016; Garner et al. 2024; Blagoderov et al. 2017). The digitised data has been made publicly accessible through the NHM data portal, offering valuable resources for researchers, conservationists, and the public.

Our research is focused on developing an advanced image classification pipeline specifically engineered to identify incorrectly labelled specimens at the species level within the iCollections. Utilising our pipeline, we can detect instances where specimens, presently labelled as one species, are consistently predicted by the system to belong to a different species. These flagged specimens are then organised and presented for a streamlined visual verification process by collection staff. In scenarios where a definitive determination remains inconclusive, we integrate more traditional methods such as reviewing ecological data associated with specimens (sample location, collector and/or the geographic range of specimen) and when a conclusive answer is unable to be obtained, we utilised molecular methods to ascertain final verification. This blend of AI‐driven analysis and more traditional techniques not only streamlines the verification process but also significantly contributes to the integrity and reliability of NHCs in the ever‐evolving landscape of biodiversity research.

## 2 Methodology

### 2.1 Data Set Creation and Image Preprocessing

The iCollections dataset comprises the British and Irish Lepidoptera (Lepidoptera Linnaeus, 1758) collections housed at the NHM. We split the collection into the butterflies and moths. Both groups were filtered to only include species where the total number of specimens was equal to or greater than 400 per species, allowing for a sufficient number to train (250 images), validate (50 images) and run inference with the remaining images (≥ 100). Low numbers of training specimens have been shown to result in poor CV performance (Xu et al. 2023; Buslaev et al. 2020; Shorten and Khoshgoftaar 2019). The filtered butterfly dataset comprised 59 species and a total of 127,671 individual specimens, while the moth dataset comprised 283 species, with a total of 222,537 individual specimens. Both training and validation images were synthetically augmented four times by the application of rotations, zooms and slight brightening, thereby generating varied synthetic images; augmenting datasets in this manner has been shown to enhance CV performance (Shorten and Khoshgoftaar 2019; Khalifa et al. 2022).

### 2.2 Model Architecture and Training Procedure

We utilised a VGG16 (Simonyan and Zisserman 2014) base with a custom selection of top layers, totalling 26 layers. This model used the ImageNet weights for the initial foundational learning, leveraging the pre‐existing knowledge embedded within the base model. In the initial phase of training, the VGG16 base was maintained in a locked state, focusing the learning process on the custom top layers for a duration of five training runs. Then for the fine‐tuning phase, the remaining layers were unlocked except for the bottom eight layers. This was allowed to run indefinitely but had a strict ‘early stopping’ protocol that would cease training after 1 decrease in the validation accuracy score and would save the best weights once finished. Furthermore, the hyperparameters of the custom top layers of the model were optimised using the ‘TF‐keras‐tuner’ library. The resultant optimum values obtained from this process were consistently applied across all runs and across both moths and butterflies, ensuring uniformity and precision in our approach. Additionally, all model runs were seeded with the same value to ensure reproducibility and to initialise each model with identical starting parameters and neural network weights. This would also mean that when a respective trained model is used for inference, it will always give the same prediction results.

### 2.3 Dataset Cropping

Initial trials of the dataset and model architecture employed a heatmap‐based class activation mapping (CAM) system to verify that the neural network within the trained model utilised features upon the specimens rather than to irrelevant background noise. The ‘GradCAM’ system was selected for this purpose because it can visualise the pixels and regions that contribute most strongly to the prediction of the model by scoring pixels and overlaying a heat‐map colour system based on this score (Selvaraju et al. 2020). Hollister et al. (2023) showed that properly trained CV models combined with heat‐maps can highlight the morphological features that distinguish closely related species.

**FIGURE 1:**
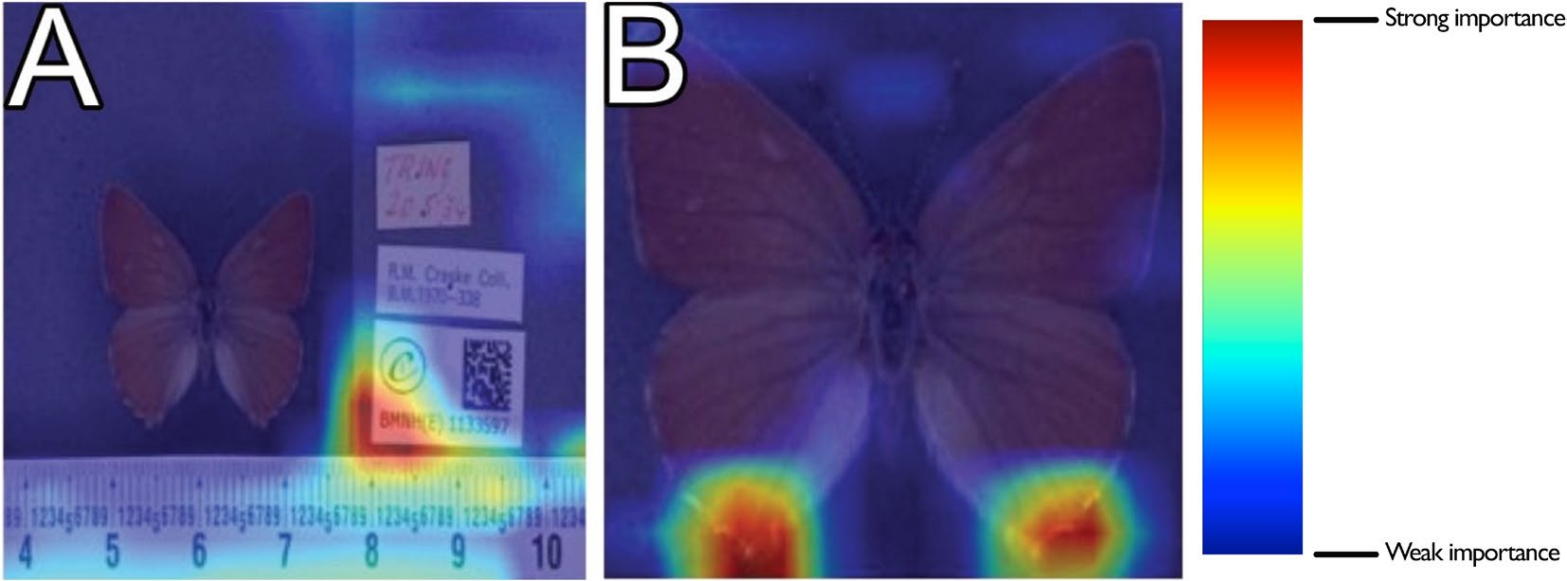
Example of heat‐map attention on labels (A) versus directly on the specimen (B).

During preliminary tests, many heat‐maps concentrated on the specimen labels instead of the insects themselves (Figure 1A). To mitigate this, we implemented a separate preprocessing pipeline using the YOLOv8 object detection algorithm trained specifically to detect Lepidoptera specimens (Sohan et al. 2024). The pipeline crops each image using the bounding boxes returned during inference, thereby excluding most irrelevant background. Subsequent heatmap analysis of these cropped images showed that a model's attention was now appropriately focused on the specimens rather than on the labels (Figure 1B).

### 2.4 Pipeline Development

**FIGURE 2:**
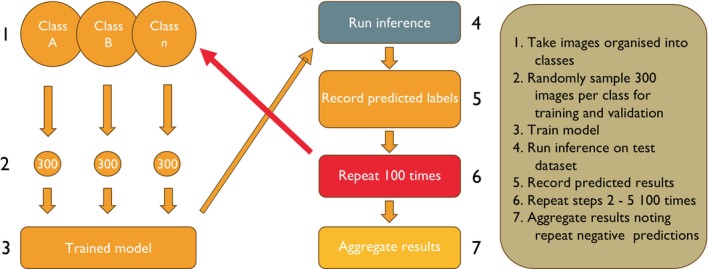
Flow diagram showing the pipeline process.

Our pipeline identifying specimens that do not match their labelled species status is shown in Figure 2. The butterfly dataset comprised 59 species and the moth dataset 283 species, where each species is one class (step 1). For every run of the pipeline, 300 images were sampled at random from the full set of images for each class, of which 250 were reserved for training and 50 for validation (step 2). Then the images were augmented, and the model was trained and validated (step 3). All remaining images (109,971 butterfly and 137,637 moth) create the test set. The trained model then performed inference on the entire test dataset using TensorFlow's *Evaluation* protocol. This assigned each test image the label with the highest confidence score and compared it with the species label it is currently assigned to determine whether the prediction was correct (step 4) and was recorded (step 5).

Steps 2–5 were repeated 100 times, each repetition sampling a fresh training and validation subset (step 6). Because the test pool vastly exceeded the training and validation pools required for an individual run, there is a probability that images appeared in the test set several times. Across the 100 runs, the number of times the pipeline classified a specimen image as a different species label from its current species label was counted. When this misclassification was found to be designated as the same label on each of the trained models, this value was noted and was designated the image's ‘Reoccurring Prediction Value’ (RPV) (step 7). For example, if the pipeline classifies a specimen as species A for each of the 100 pipeline runs, but its current species label identifies it as species B, then it is assigned a RPV of 100.

### 2.5 Human Interrogation

Taxonomists specialising in morphological identification of Lepidoptera from the NHM, with a combined expertise spanning over 50 years, were enlisted to help inspect specimens flagged by the model. Specifically, they were tasked with looking at specimens that the model identified as belonging to a species that is different from its current NHM species record. They were tasked with visually inspecting specimens that were flagged by the pipeline from within the NHM collections. They were told to verify specimens according to four options:

1. Labelled wrong: The specimen was incorrectly labelled in the collection.
2. Pipeline wrong: The pipeline made a mistake and incorrectly predicted a specimen as a different species to that which it was labelled as in the collection.
3. Portal wrong: The specimen was correctly labelled in the collection; however, it was incorrectly labelled as the wrong species (or not present) upon the NHM data portal.
4. Unknown: The experts were unable to verify what the specimen was or that it was currently inaccessible.

They also added notes to each specimen examined, noting what could have resulted in either of the four choices. To visually inspect every specimen across the two groups would have taken a very long time for the small team of experts. Therefore, it was decided to go through a sample of the specimens with RPVs > 80, allowing for a review of the most likely mislabelled specimens. Additional specimens with RPVs < 80 were also examined. The examinations were conducted over 4 sessions with an allotted time of 16 h. This resulted in a total of 210 specimens being examined.

### 2.6 Note Standardisation

Notes and comments were standardised. Each specimen was assigned a visual‐difficulty score as follows:

1. Easy to verify with the naked eye.
2. Difficult, but not impossible, to verify.
3. Difficult; required additional contextual information (e.g., sampling location, date, or size relative to the predicted species).
4. Impossible to verify visually; referred for further confirmation.

### 2.7 Genetic Verification

Specimens unable to be verified visually (category 4 above) were designated for genetic verification. However, several additional specimens not in this category were selected to allow for validation of the visual based verification conducted by the experts. DNA was extracted in a dedicated historical DNA facility using the protocol outlined by Hall et al. (2023), with NGS library building following the protocol detailed in Marsh et al. (2025), using the ‘Santa Cruz Reaction’ (Kapp et al. 2021) with the modifications of Nguyen et al. (2023). Libraries were shotgun sequenced on an Illumina NovaSeq XPlus 25B lane with a commercial provider, targeting 5–10 million PE reads per specimen. The COX1 barcode gene was recovered using MitoGeneExtractor (Brasseur et al. 2023) which uses exonerate (Slater and Birney 2005) to map reads to a target reference, in this case the closest reference sequence available on NCBI protein database along with ~40 common contaminant sequences (i.e., bacteria, fungi, human, wolbachia) to help filter out non‐target reads.

## 3 Results

### 3.1 Pipeline Results

The 100 butterfly model runs achieved a range of F1‐scores between 0.9497 and 0.9267 and the 100 moth model runs achieved a range of F1‐scores between 0.8486 and 0.8386. The F1‐score is the harmonic mean of precision and recall, and provides a balanced measure of classification performance. Out of the original 127,671 butterfly specimens, 17,562 individual specimens were flagged by the model at least once across all 100 runs. The number of specimens that received a RPV of one greatly outnumbers the number of specimens that received a RPV of 100 (Table 1). When the RPV are combined into intervals of 10, over 83% of specimens are categorised with an RPV of 1–10, with the next interval of 11–20, occurring over 6%. Less than 1% of specimens flagged by the pipeline occurred in the RPV interval of 91–100. Out of the original 222,537 moth specimens, 81,788 individual specimens were flagged by the model at least once across all 100 runs. Again, the number of specimens that received a RPV once outnumbers the specimens that were received a RPV of 100 (Table 1). Over 80% of specimens flagged by the pipeline occurred in the RPV interval of 1–10, with the next interval of 11–20, occurring over 9%. Just over 0.1% of specimens occurred in the interval with a RPV of 91–100.

**TABLE 1:** Reoccurring prediction values (RPV) for butterflies and moths in intervals of 10.

RPV interval	Butterfly: number of specimens	Butterfly: percentage of total	Moth: number of specimens	Moth: percentage of total
91–100	171	0.97	94	0.11
81–90	157	0.89	255	0.31
71–80	129	0.73	255	0.43
61–70	121	0.69	446	0.55
51–60	179	1.02	613	0.75
41–50	242	1.38	1088	1.33
31–40	295	1.68	1834	2.24
21–30	507	2.29	3307	4.04
11–29	1121	6.38	8150	9.96
01–10	14,639	83.36	65,652	80.27

### 3.2 Visual Verification Interrogation

#### 3.2.1 Error Type Analysis

In total, 210 specimens were visually inspected: 120 butterflies and 90 moths. 56.67% of the specimens examined had an RPV > 80, meaning that they were consistently flagged by the model as being incorrectly labelled (Figure 5). An additional 493 hybrid butterflies were flagged by the pipeline; however, these technically belong to no official species and were verified to be hybrids by the experts, and these were excluded from the remaining evaluations.

**FIGURE 3:**
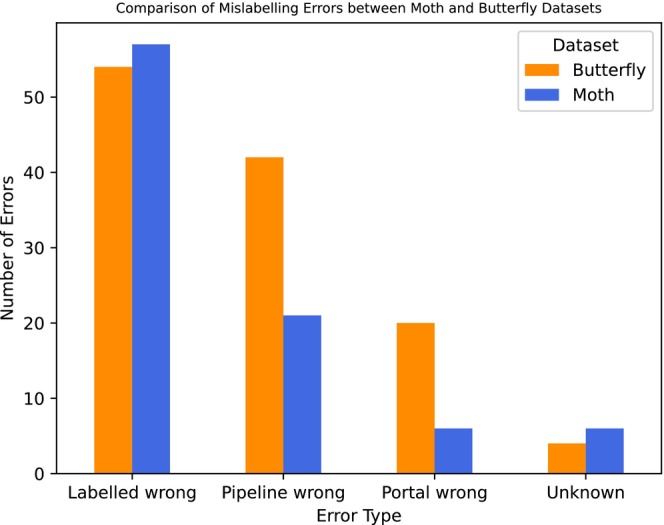
Bar chart showing the combined error results for the butterflies and moths.

The most commonly occurring error among the specimens that were visually inspected by the taxonomists was that the specimens were labelled wrong (54 butterfly, 57 moth) (Figure 3). This was followed by the pipeline being wrong (42 butterfly, 21 moth), then the portal being wrong (20 butterfly, 6 moth), with the lowest category being unknown (4 butterfly, 6 moth).

#### 3.2.2 Difficulty of Verification Analysis

**FIGURE 4:**
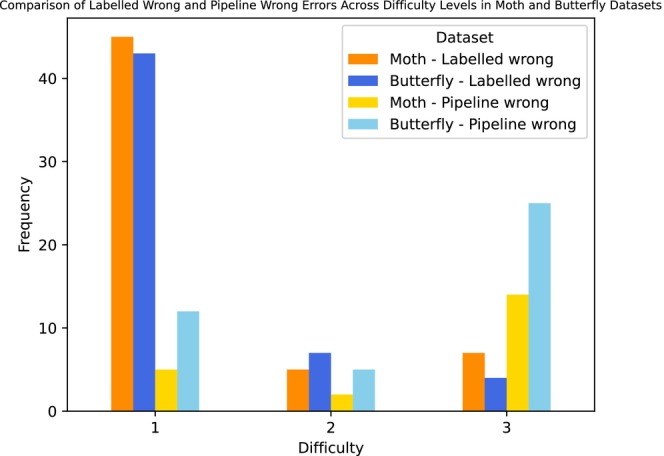
Bar chart showing the difficulty assigned to the visual verifications for moth and butterfly specimens.

In general, specimens that were given a difficulty score of 1 by the taxonomists were more likely to be labelled wrong (Figure 4). This pattern is seen in reverse when examining verifications with a difficulty score of 3, where the pipeline was more often the reason for the errors. This demonstrates that errors in the labelled wrong category were more likely to be rated as easy to visually verify (score of 1), while errors in the pipeline wrong category were more likely to be rated as difficult to visually verify (score of 3).

#### 3.2.3 Relationship Between Difficulty and RPV

**FIGURE 5:**
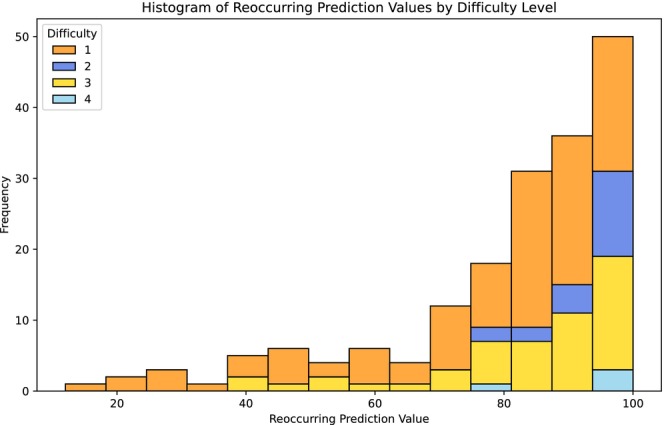
Histogram showing the reoccurring prediction value and difficulty of specimens visually examined.

Most specimens examined had high RPV values, but in general, as RPV decreases, the difficulty level also tends to decrease (Figure 5). Difficulty Level 1, which contains the most specimens, shows the greatest variability, with prediction values distributed across the entire range. In contrast, Difficulty Levels 3 and 4 are more prevalent among specimens with higher RPVs.

#### 3.2.4 Examples of Verified Labelled Wrong Specimens

**FIGURE 6:**
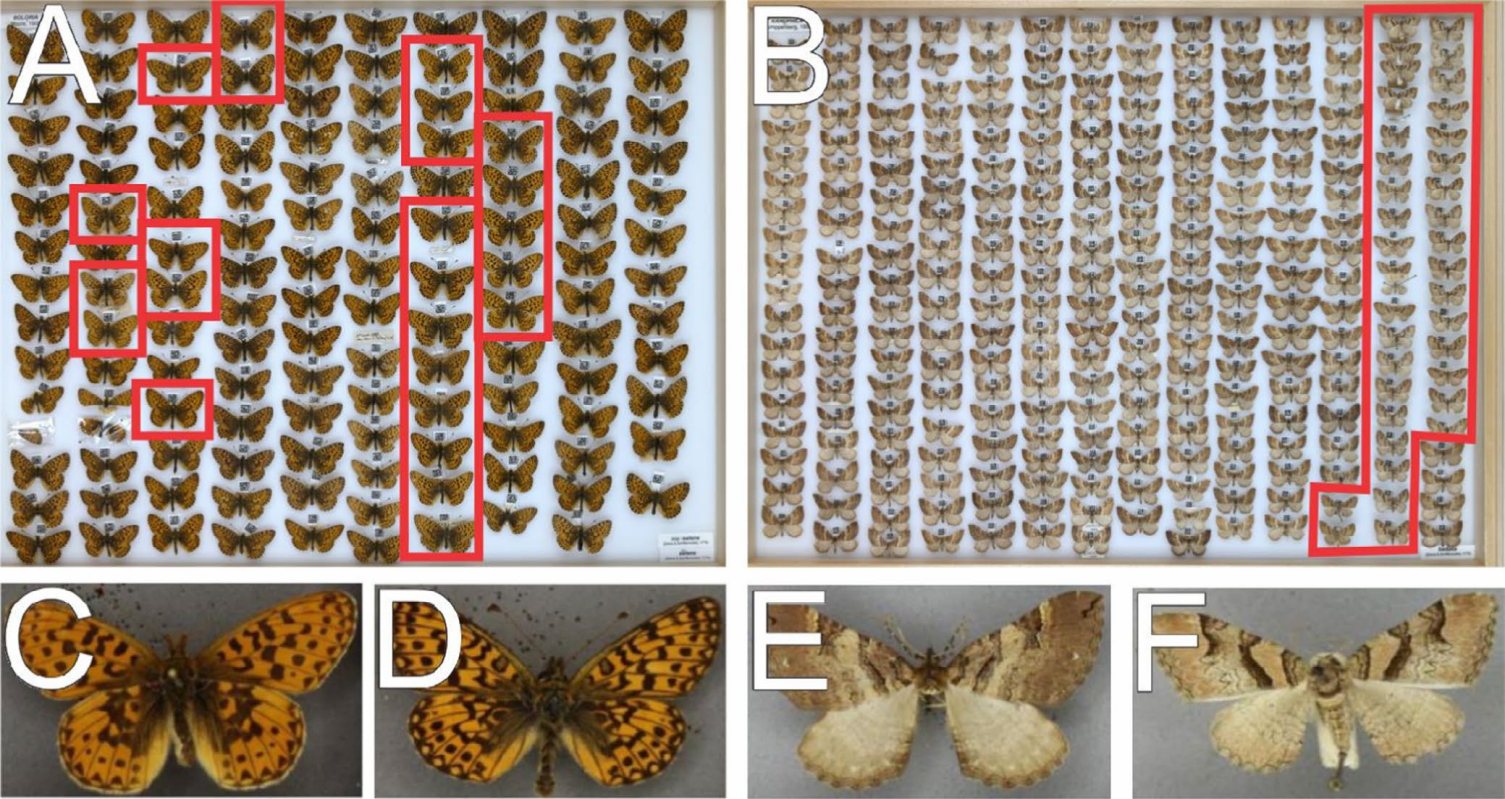
Verified example of labelled wrong specimens flagged by the pipeline. Whole drawer image of Boloria selene (A) with an example specimen of this species (C). Highlighted specimens in (A) are Boloria euphrosyne (D) but mislabelled as B. selene. (B) is a whole drawer of Earophila badiata with an example specimen of this species (E). Highlighted specimens in (B) are Cupido minimus (F) but mislabelled as E. badiata..

Here we present two examples of when labelling was incorrect. Figure 6A is a whole drawer of *Boloria selene* (Denis & Schiffermüller, 1775) (Figure 6C) while those highlighted are *Boloria euphrosyne* (Linnaeus, 1758) (Figure 6D). Guides dedicated to visual morphology separate these two species based on the pattern of the outside edges of the wings with little else considered to separate specimens (European Butterflies Group 2024). However, once the difference was noted, experts found it easy to discern between the two and gave these a difficulty of 1. Figure 6B is a whole drawer image of *Earophila badiata* (Denis & Schiffermüller, 1775) (Figure 6E) while the highlighted specimens are of *Catarhoe rubidata* (Denis & Schiffermüller, 1775) (Figure 6F). Visual verification of these specimens was, in the opinion of the experts, easy to discern and gave these a difficulty of 1. Moreover, these specimens were all input by a single curator and again, according to the experts, it was a mistake that should have been avoided.

#### 3.2.5 Examples of Verified Pipeline Wrong Specimens

**FIGURE 7:**
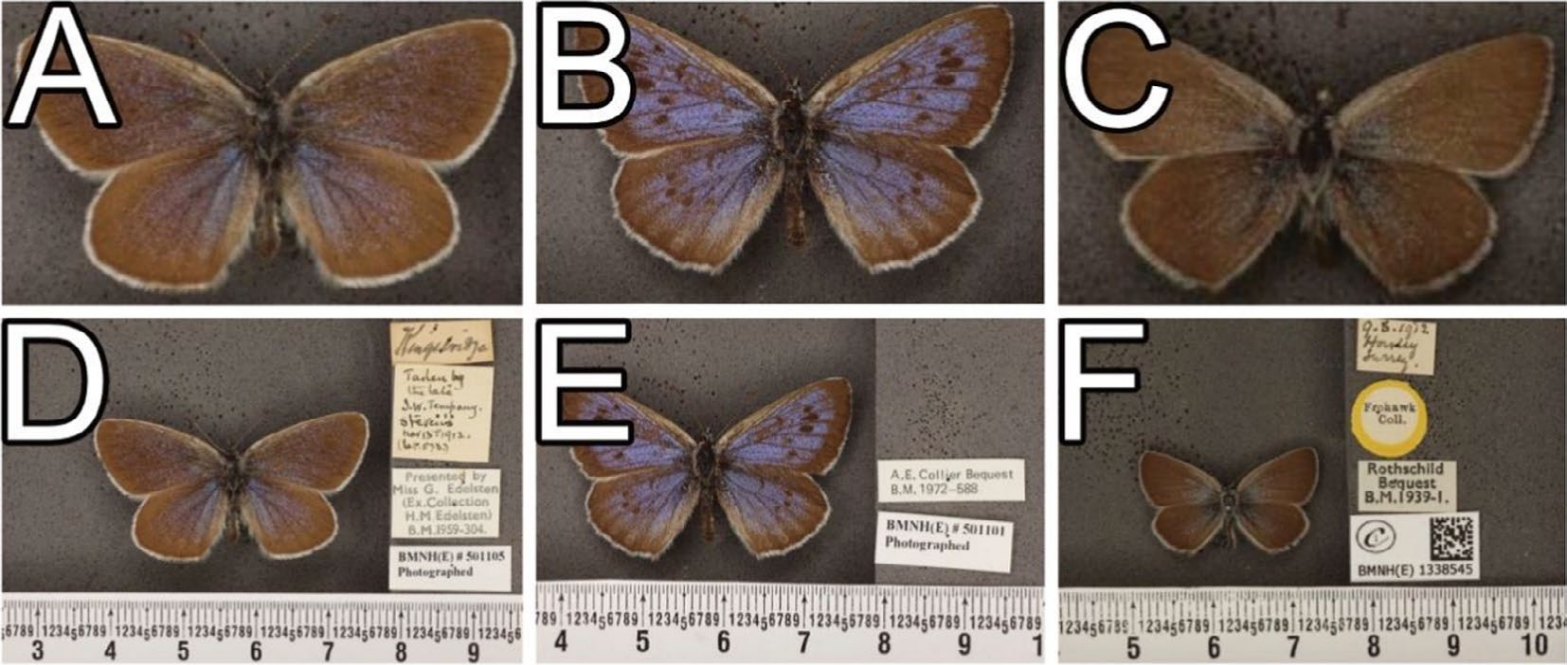
Verified example of when the pipeline made an incorrect prediction. Specimen ‘BMNH(E)_501105’ (A, D) is correctly labelled as *Maculinea arion* on the specimen label (B, E), but was incorrectly predicted by the pipeline to be *Cupido minimus* (C, F). Although the visual taxonomists note that these species look similar when images are cropped without scale bars (A–C), the size differences between these species are obvious traits that are used to tell them apart (D–F).

Specimen ‘BMNH(E)501105’ (Figure 7A) belongs to the species *Maculinea arion* (Linnaeus, 1758) (Figure 7B). The pipeline predicted this specimen as *Cupido minimus* (Fuessly, 1775) (Figure 7C) with an RPV of 93. Visual verification by the experts confirmed that the pipeline labelled this wrong due to a large size difference between the current species label and predicted species label as can be seen in the images with scalebars and labels (Figure 7D–F). The experts noted that while the morphology when viewing the cropped images does resemble the predicted species, the specimen in question could easily be verified when viewing it in person or when viewing the image alongside the scalebar.

**FIGURE 8:**
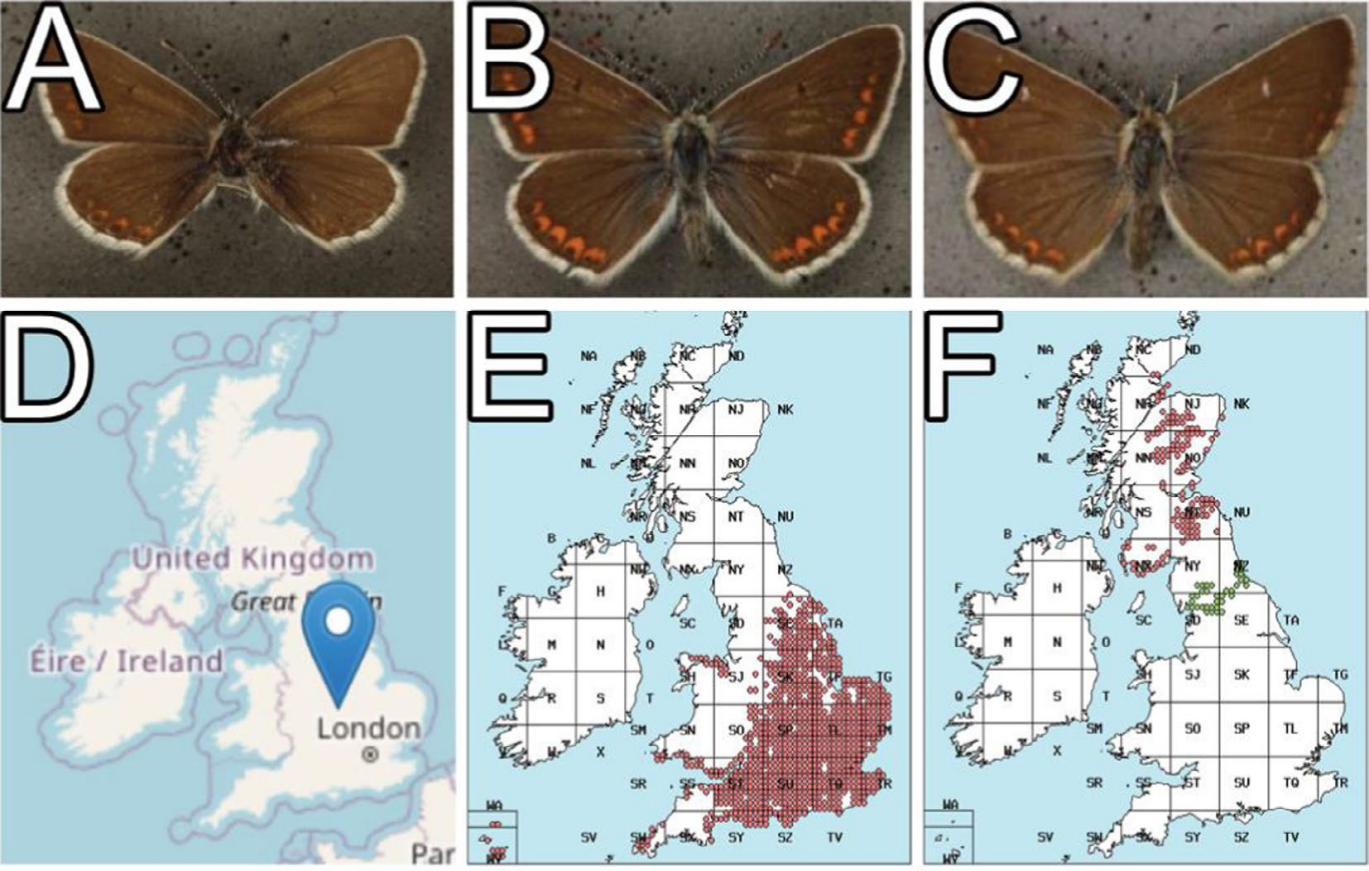
Verified example of when the pipeline made an incorrect prediction. Specimen ‘BMNH(E)1390409’ (A) its sampled location (D) with an example of this species *Aricia agestis* (B) and collection locations for this species (E). The pipeline incorrectly predicted this specimen to be Aricia artaxerxes (C), but it is only found in the northern portion of the UK (F) and does not overlap with A. agestis.

Figure 8A shows specimen ‘BMNH(E)1390409’ belonging to *Aricia agestis* (Denis & Schiffermüller, 1775) (Figure 8B). The pipeline predicted this as *Aricia artaxerxes* (Fabricius, 1775) with an RPV of 98 (Figure 8C). Visual verification confirmed that the pipeline had labelled this wrong because the location that the specimen was sampled from was outside its geographic range. Again, it was noted that while the morphology of the specimen in question resembled the predicted species rather than actual species, the location that the specimen was sampled from would verify that the pipeline predicted it incorrectly. Figure 8D is the location the specimen was sampled from while Figure 8E is the range of the current species label and Figure 8F is the range of the predicted species label.

#### 3.2.6 Examples of Portal Wrong

**FIGURE 9:**
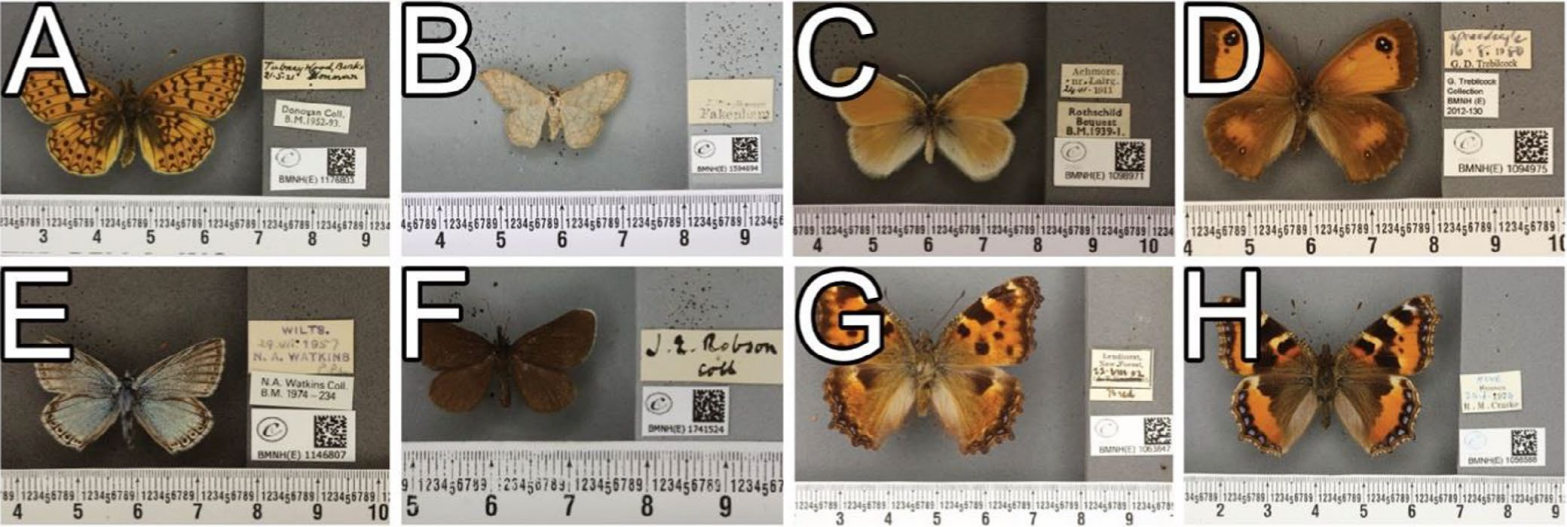
(A–H) Various examples of issues with specimen storage and retrieval from within the NHM portal.

Figure 9 highlights various errors on the NHM portal in which specimens, their associated information, or their retrieval via the search function from the server storage can be affected. When ID BMNH(E)1176803 (Figure 9A) is requested on the portal, links for two specimens are retrieved (Figure 9A,B). When ID BMNH(E)1098971 is requested, a single link is retrieved that contains two specimens (Figure 9C,D). Although the ID number matches the ID on specimen 8C, the information on the link belongs to specimen 8D, yet the ID on 8D is different (1094975). Further complicating the mislabelling, specimen 9C, *Coenonympha tullia* (Müller, 1764), is not of the same family as specimen 8D, *Pyronia tithonus* (Linnaeus, 1758). When searching for ID BMNH(E)1146807 (Figure 9E), the portal retrieves a completely different ID, and the associated information belongs to specimen 9F. When ID BMNH(E)1063847 is requested, a link for specimen 9G is retrieved. Upon reviewing the information on this link, although the ID number matches the specimen, the attached information (i.e., its taxonomic name, its sampling coordinates, and its drawer number within the collections) belongs to a different species (Figure 9H).

### 3.3 Genetic Verification Results

**FIGURE 10:**
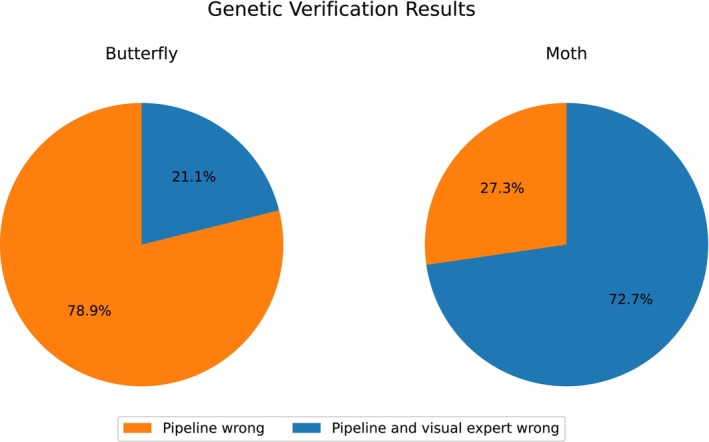
Pie charts showing the results from the genetic verification.

Thirty specimens were selected for genetic analysis, made up of 19 butterfly specimens and 11 moth specimens (Figure 10). Among the butterflies, 15 specimens that the visual taxonomists had flagged as incorrectly identified by the pipeline were confirmed as incorrect through genetic analysis. However, four specimens contradicted both the pipeline's prediction and the visual taxonomists' assessment, which had supported the pipeline's prediction. For the moths, genetic analysis confirmed that three specimens were incorrectly identified by the pipeline, in line with the visual taxonomists' assessment. In contrast, the genetic analysis showed that eight specimens contradicted both the pipeline and the visual taxonomists. Out of the four specimens that were given a difficulty score of four (three butterflies and one moth), only two came back from the genetics examination. Both contradicted the pipeline's predictions.

## 4 Discussion

A primary challenge of natural history museums is the taxonomic identification, curation and management of vast and continuously growing numbers of specimens (Miller et al. 2004; Mujtaba et al. 2018). Our study describes a CV pipeline applied to the digitised British and Irish Lepidoptera collection at the NHM, London. This pipeline was developed to automatically identify mislabelled specimens, thereby enhancing the accuracy and efficiency of managing these collections.

Out of the original 127,671 individual butterfly specimens, 17,562 were flagged by the pipeline at least once (out of 100 runs, 28.37%). However, analysis of RPV demonstrated that > 83% of specimens received an RPV of 1–10, while less than 1% reached an RPV of 91–100. Similarly, for the moth dataset comprising 222,537 specimens, 81,788 were flagged by the pipeline at least once, with over 80% falling within the RPV range of 1–10, while less than 0.12% received an RPV of 91–100. This suggests that while many specimens were flagged by the pipeline, only a small fraction were consistently flagged as being potentially mislabelled. Such specimens (e.g., RPV > 80) should be visually inspected by taxonomists and re‐labelled if required. This is supported by expert visual examination where 56.67% of the specimens they examine had an RPV > 80 (Figure 5). We confirmed that 147 of the inspected specimens (out of 210; 70%) were indeed incorrectly labelled, either within the collection or during the digitisation process.

In contrast, specimens within the lower RPV ranges (i.e., 1–10) will most likely show pipeline‐based errors due to the dataset containing mislabelled specimen images. Although only a small portion of the total number of flagged specimens was examined, 70% of those examined were incorrectly labelled as either labelled wrong or portal wrong. Even in a scenario where these specimens were the only mislabelled specimens within the dataset, they would ultimately destabilise a CV model's true potential. Research has shown that incorrectly labelled specimens that have been used in the training dataset erode the accuracy of the resulting model (Northcutt et al. 2021). Therefore, it could be assumed that a model known to have incorrectly labelled specimens will undoubtedly produce false positive predictions. Future work should focus on this and investigate whether there is a relationship between RPV values and pipeline accuracy.

The mislabelled specimens identified in this study underscore the complexity of managing and curating large NHCs. Our findings align with previous research suggesting that manual labelling errors are not uncommon in such extensive collections, with errors reported to be as high as 50% within certain collections (Goodwin et al. 2015). This substantial error rate highlights the critical need for technological solutions (such as that described here) to be used in combination with expert knowledge for the curation and maintenance of large NHCs. Here, we show that automated methods can be used to flag specimens that are potentially labelled differently from their current status. However, in order to verify and rectify such curation issues, the expert opinion and extensive knowledge of museum curators and taxonomists are needed. We see the collaboration between automated methods and traditional taxonomists as being key for the future curation and maintenance of very large and growing NHCs.

The genetic analysis confirmed that the pipeline made several incorrect predictions, highlighting areas where it aligned with human expertise and also contradicted their predictions. For the butterfly specimens, 15 instances were identified where the pipeline predictions were incorrect, and these errors were accurately caught by the visual taxonomists, showcasing their taxonomic expertise. However, in four cases, the genetic analysis contradicted both the pipeline and the visual taxonomists, indicating that both methods occasionally fail to capture the true identity of certain specimens. Similarly, for the moth specimens, three cases were confirmed where the pipeline predictions were incorrect, and these errors were also identified by the visual taxonomists. In contrast, eight instances showed that the genetic analysis went against the predictions of both the pipeline and the visual taxonomists. These findings suggest that while the pipeline can be effective in identifying potential mislabelling, it is not infallible, reinforcing the importance of a multi‐faceted approach to specimen verification.

The synergy of CV, visual and genetic methods offers robust approaches for managing and curating large NHCs. The combination of these methods is particularly important given the challenges associated with each. Visual verification can be subjective and dependent on the availability and expertise of taxonomic specialists (Austen et al. 2016), while genetic analysis, though precise, can be resource‐intensive and sometimes impractical for older or degraded specimens (Karbstein et al. 2024).

Despite the promising results, our study has several limitations. One notable limitation is the current inability of the pipeline to integrate information on specimen size differences or geographical range. For instance, some species may be morphologically similar but vary significantly in size or are endemic to different regions, leading to potential misidentifications by the pipeline (Figure 7 and 8). Initial testing showed that the original images which included scalebars and labels interfered with the training of the CV models and resulted in the models occasionally utilising these parts of the images rather than the desired specimen. This was circumvented by cropping the images so that the specimens took up as much of the image as possible, resulting in reduced noise for model training. However, this ultimately resulted in the pipeline unable to differentiate between size as all images are processed as the same size. This limitation suggests that further refinement of the pipeline is necessary to incorporate additional contextual data, such as specimen size and collection location, to improve accuracy. Additionally, experimenting with systems where CV models focus on specific areas while ignoring excessive noise could be explored.

Our results have revealed a wide range of reasons why specimens within NHCs can become mislabelled, with the biggest being human error. Some specimens showed clear and obvious morphological defining features that should have been, at least in the opinion of the visual‐based experts, easy to have been correctly labelled. Due to the age of some of these collections (Paterson et al. 2016), the true reasons as to how these errors occurred will never be known. However, current issues where limitations in resources mean that curation staff are unable to dedicate sufficient time to manually verify specimens and manage collections mean that these errors could persist. Specimens that are mislabelled on the portal can also be attributed to human error. The journey of a specimen from its initial input into the collections to its eventual digital representation on the portal would have gone through many different individuals including several generations of curators, photographing teams, or server‐level teams, all with varying levels of expertise. The NHM is currently several years into an ambitious project to digitise and upload their NHCs. This highlights that communications from different departments should be a priority when creating such projects and implementing verification steps to avoid errors.

Our study demonstrates that automated methods can be used as important tools for taxonomists and curators to manage very large NHCs. Future work should focus on developing user‐friendly interfaces and tools for museum staff and taxonomists to easily interact with and validate the results from the CV pipeline, which could streamline the verification process and free up staff time for other collection management tasks and research.

## 5 Conclusion

In conclusion, our study demonstrates the potential of a combined approach using CV, visual verification, and genetic analysis to significantly improve the accuracy and efficiency of managing NHCs. By automating the initial identification of potentially mislabelled specimens, our CV pipeline offers a scalable solution to the pervasive issue of taxonomic misidentification in large collections. This automation not only enhances the speed and accuracy of specimen verification but also alleviates the burden on human experts, allowing them to focus on more complex tasks that require specialised knowledge.

The integration of AI‐driven technologies into museum curation practices represents a significant step forward in preserving the integrity and utility of these invaluable scientific resources for future research and conservation efforts. Furthermore, our approach underscores the importance of a multi‐faceted verification process, combining the strengths of various methodologies to achieve a more reliable and comprehensive system. By continuing to innovate and improve these methods, we can ensure that natural history collections remain accurate, accessible, and valuable resources for scientists and researchers worldwide, thereby supporting ongoing biodiversity research and conservation initiatives.

## Author Contributions

**Jack D. Hollister:** conceptualization (lead), data curation (lead), formal analysis (lead), methodology (lead), writing – original draft (lead), writing – review and editing (lead). **Geoff Martin:** data curation (supporting), formal analysis (supporting). **Xiaohao Cai:** supervision (supporting), writing – review and editing (supporting). **Tammy Horton:** supervision (supporting), writing – review and editing (supporting). **Owain Powell:** formal analysis (supporting). **Mark Sterling:** formal analysis (supporting). **Glory Turnbull:** formal analysis (supporting). **Ben W. Price:** data curation (supporting), formal analysis (supporting), supervision (supporting), writing – review and editing (supporting). **Phillip B. Fenberg:** conceptualization (supporting), data curation (supporting), formal analysis (supporting), methodology (supporting), supervision (lead), writing – original draft (supporting), writing – review and editing (supporting).

## Conflicts of Interest

The authors declare no conflicts of interest.

## Data Availability

The code associated with this project can be found at: https://github.com/JackDanHollister/Finding_mislabelled_specimens_in_NHCs. The image set used can be downloaded from the NHM, London portal: https://data.nhm.ac.uk/.
